# Aortic Valve Infective Endocarditis Complicated by Annular Abscess: Antibiotics in the Abscess Cavity

**DOI:** 10.3390/jcdd11070189

**Published:** 2024-06-24

**Authors:** Zaki Haidari, Shehla Ufaq Ahmad, Stephan Knipp, Iskandar Turaev, Mohamed El Gabry

**Affiliations:** Department of Thoracic and Cardiovascular Surgery, West German Heart and Vascular Center Essen, University Hospital Essen, 45147 Essen, Germanyiskandar.turaev@uk-essen.de (I.T.);

**Keywords:** infective endocarditis, aortic valve, annular abscess, patch

## Abstract

Objectives: Infective endocarditis of the aortic valve complicated by annular abscess is a challenging problem and often requires patch reconstruction after surgical debridement of the abscess cavity. Filling the remaining cavity with antibiotics is advocated to prevent recurrent endocarditis. This study aimed at evaluating the role of local antibiotics in patients with aortic valve infective endocarditis complicated by annular abscess. Methods: Between January 2012 and December 2021, all consecutive patients with aortic valve infective endocarditis complicated by annular abscess undergoing cardiac surgery and annular patch reconstruction were included. Patients receiving local antibiotics were compared with patients without local antibiotics. The primary endpoints were the incidence of recurrent endocarditis, re-operation, and mortality during two-year follow-up. Results: A total of 41 patients with aortic valve infective endocarditis complicated by annular abscess underwent surgical patch reconstruction after radical debridement. In total, 20 patients received local antibiotics in the abscess cavity and 21 patients were treated without local antibiotics. The most common causative microorganisms were the staphylococci species and the most common location of the abscess was the non-coronary annulus. During two-year follow-up, one patient in each group developed recurrent endocarditis (*p* > 0.99) and both patients were reoperated (*p* > 0.99). Two-year mortality was 30% in the local antibiotic group and 24% in the control group (*p* = 0.65). Conclusions: Radical debridement and patch reconstruction of the aortic annulus in patients with aortic valve infective endocarditis complicated by annular abscess is an effective surgical strategy. Filling of the remaining abscess cavity with antibiotic seems not to affect the rate of recurrent endocarditis, reoperation, and mortality during two-year follow-up.

## 1. Introduction

Cardiac surgery in patients with aortic valve infective endocarditis is associated with increased mortality and morbidity [1], especially in complicated cases such as annular abscess [2,3]. Radical surgical debridement of the abscess cavity is necessary to prevent recurrent endocarditis [4]. However, radical surgical debridement can result in tissue defects leading to the necessity for annular patch reconstruction to anchor a prosthetic valve. Additionally, the application of local antibiotics in the remaining abscess cavity has been proposed to prevent recurrence of infective endocarditis [5,6,7] and improve outcome [8]. In this study, we aimed to evaluate the effect of local antibiotic application in the remaining abscess cavity on clinical outcome in patients with aortic valve infective endocarditis complicated by annular abscess and a need for annular patch reconstruction.

## 2. Materials and Methods

### 2.1. Patients

Eligible candidates for this retrospective comparative study were patients with definitive infective endocarditis [9] of the aortic valve undergoing cardiac surgical therapy from January 2012 to December 2021. Out of these, 71 patients were found to have annular abscess receiving annular patch reconstruction. Annular abscess was defined as paravalvular cavity with infectious or necrotic tissue in the aortic annulus or root, or as aortoventricular discontinuity. Patients undergoing replacement of the intervalvular fibrous body were excluded. The study was reviewed and approved by the institutional ethics committee (Protocol number: 23-11446-BO, approval date: 28 September 2023) and written informed consent was waived due to retrospective study design.

### 2.2. Surgical Technique

**Figure 1:**
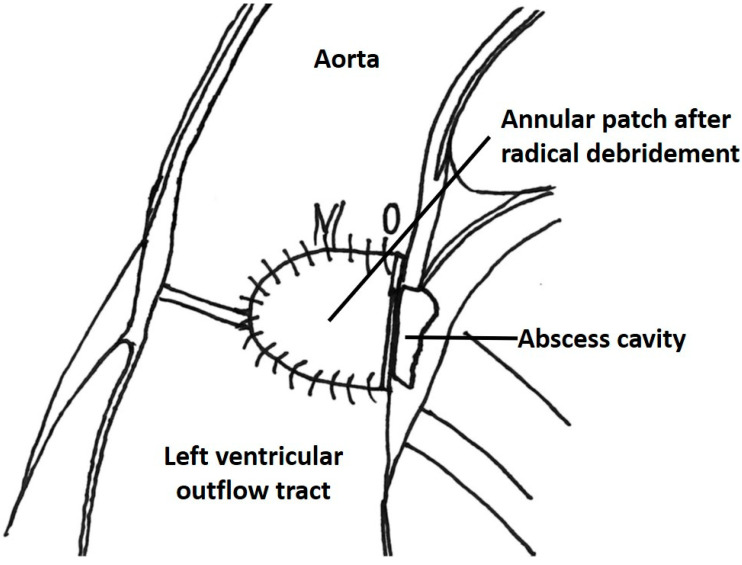
Patch reconstruction of the aortic annulus after radical debridement of the annular abscess cavity.

Standard aortic and caval cannulation techniques were applied. Cardioplegic arrest was achieved by crystalloid cardioplegia (Custodiol, Dr. Franz Koehler Chemie, Bensheim, Germany). After resection of the infected aortic valve leaflets, the aortic annulus was inspected for the presence of abscess. In cases with annular abscess, the abscess cavity was opened and all macroscopically infected tissue was removed. After irrigation with diluted povidone–iodine solution, the defect was excluded with autologous or bovine pericardial patch, respectively (Figure 1). Application of local antibiotics in the remaining abscess cavity was at the discretion of the operating surgeon. In cases with local antibiotics, the remaining cavity was filled with a mixture of antibiotic (dependent on the causative microorganism antibiogram) and fibrin glue before the closure of the defect. Finally, aortic valve replacement was performed. Concomitant procedures were performed as indicated. All instruments, synthetic material, and prostheses were soaked in vancomycin solution during operations.

### 2.3. Postoperative Care

Postoperatively, all patients were transferred to the cardiac surgical intensive care unit (ICU) with invasive hemodynamic monitoring and guideline-directed antibiotic and supportive therapy. Follow-up was conducted by outpatient visits or telephone follow-up, and data on survival and valve-related complications were collected.

### 2.4. Endpoints

The primary endpoints of the study were the incidence of recurrent infective endocarditis, reoperation, and mortality at two-year follow-up. Recurrent infective endocarditis was defined as relapse or reinfection according to guidelines. Secondary endpoints included postoperative organ failure (circulatory, pulmonary and renal), and ICU and hospital stay.

### 2.5. Statistical Analysis

SPSS software version 29 (SPSS Inc., Chicago, IL, USA) was used to analyze the data. Continuous variables were reported as median and interquartile range (IQR) and compared with the Mann–Whitney test. Categorical data were expressed as number of patients and frequencies and compared using the chi-square or Fisher exact test. Kaplan–Meier curves were employed to evaluate overall survival rates and the two groups were compared using the log-rank test. A *p*-value < 0.05 was considered statistically significant.

## 3. Results

### 3.1. Baseline Characteristics

From January 2012 through December 2021, 211 patients underwent cardiac surgery for infective endocarditis of the aortic valve. In 79 (34%) patients, an annular abscess was found by intraoperative inspection. A patch reconstruction of the aortic annulus was performed in 41 patients. In 20 patients, a mixture of antibiotics and fibrin glue was used to fill the remaining abscess cavity. In the remaining 21 patients (control group), the abscess cavity was left empty. The preoperative demographics and clinical and inflammatory status of the two groups are given in Table 1. The most commonly identified causative microorganism for infective endocarditis (Table 2) was staphylococcus aureus. The most common location of the annular abscess was at the level of the non-coronary cusp insertion (Table 3). There were no significant differences in demographics, hemodynamic, and pulmonary status, the levels of preoperative inflammatory parameters, causative microorganism or location of the abscesses between the two groups.

### 3.2. Operative Characteristics

Table 4 presents the operative characteristics of the two groups. All patients underwent at least aortic valve replacement. In the control group, three patients underwent aortic root replacement using a conduit. Concomitant valvular procedures were performed more frequently in the control group, without statistical significance. Concomitant coronary artery bypass grafting was performed in six patients, four in the local antibiotics group, and two in the control group (*p* = 0.41). Cardiopulmonary bypass and aortic cross-clamp times were longer in the local antibiotics group but did not reach statistical significance.

### 3.3. Endpoints

**Figure 2:**
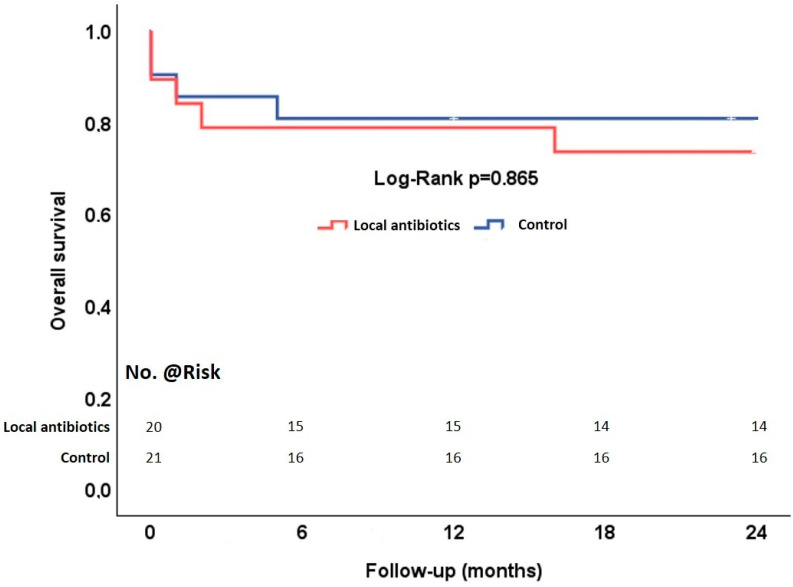
Overall survival of patients with aortic valve infective endocarditis complicated by annular abscess undergoing annular patch reconstruction with local antibiotics and control.

The endpoints are summarized in Table 5. Thirty-day mortality was 12% overall, 15% in the local antibiotics group, and 10% in the control group (*p* = 0.66). The causes of early mortality were septic multiorgan failure in three cases and cardiac failure in two cases. Two-year mortality increased to 27% overall, 30% in the local antibiotics group, and 24% in the control group (Figure 2). Recurrent infective endocarditis occurred in one patient in the local antibiotics group and in one patient in the control group (*p* > 0.99). Both patients underwent reoperation. The time interval between the index operation and the recurrence of infective endocarditis in the local antibiotics group was 19 months and 40 days in the control group.

The incidence of postoperative organ failure (circulatory, respiratory and renal) requiring (temporary) support or replacement did not differ between the two groups. There were also no differences in the ICU and hospital stay between the groups.

## 4. Discussion

Annular abscess remains a frequent complication of aortic valve infective endocarditis and requires surgical debridement. The subsequent tissue defect often requires patch reconstruction to anchor the valve prosthesis and to exclude the remaining cavity from the circulation. In this study, we evaluated the effect of applying local antibiotics in the remaining abscess cavity on outcome, especially on the rate of recurrent infective endocarditis. We found that the rate of recurrent infective endocarditis is low after radical surgical debridement and annular patch reconstruction and that the application of local antibiotics did not affect the recurrence rate or survival during two-year follow-up.

Annular abscess is strictly a consequence of an uncontrolled infection causing a mycotic aneurysm of the sinuses of Valsalva. Uncontrolled infection can also result in fistulous communication between the aortic root and the left ventricular outflow tract (paravalvular aortic regurgitation) or to other cardiac chambers such as the right atrium and the right ventricle (Gerbode defect). Suspicion of abscess formation should rise with regards to any patient with aortic valve endocarditis on guideline-directed antibiotics and persistent fever, elevated inflammatory parameters, or cutaneous or embolic phenomena. Other signs of annular abscess include prolongation of PR interval or development of complete heart block. Multimodality imaging plays an important role when evaluating a patient with aortic valve infective endocarditis complicated by annular abscess [10].

The prevalence of aortic valve infective endocarditis complicated by annular abscess varies between 12% and 50% [11,12,13]. Abscess formation is an independent predictor of short-term mortality and is reported to be twice as high compared to patients without annular abscess [1]. Furthermore, recurrent infective endocarditis is higher in patients with annular abscess compared to patients without annular abscess [14]. Therefore, it is of crucial importance to eradicate infection by radical surgical debridement and exclusion by patch reconstruction. The addition of local antibiotics in the remaining abscess cavity has been suggested to prevent recurrence. In addition, the risk of residual infected tissue seems to be low after radical surgical debridement. Furthermore, a mixture of antibiotics with fibrin glue in the remaining excluded abscess cavity may only provide temporary protection against reactivation of infection. Moreover, the presence of fibrin glue may impede the penetration of antibiotics in the surrounding tissue, mitigating the effect of such therapy. Although local antibiotics showed effectivity in the prevention of sternal wound infection [15], development of antibiotic-eluting or bacterial-resistant patches and valvular prosthesis may be a more effective strategy in the prevention of recurrent infective endocarditis in this high-risk population [16,17,18,19].

In this study, we selected only patients in whom a patch reconstruction was performed. Patients with small annular abscesses were treated by direct closure of the abscess cavity. The cohort comprises high-risk patients with large annular abscesses, who required patch reconstruction. The control group included three patients with additional aortic root replacement in which local antibiotics could not be applied. Exclusion of these patients did not change the outcome. Therefore, we decided not to exclude these patients from the analysis.

## 5. Limitations

To our best knowledge, this is the first study evaluating the role of local antibiotics in aortic valve infective endocarditis complicated by annular abscess. Although the baseline demographics, clinical and inflammatory status and operative characteristics of the two groups were comparable, bias cannot completely be excluded due to retrospective study design and limited sample size. Therefore, larger and randomized controlled studies are required before definitive conclusions can be drawn.

## 6. Conclusions

Local antibiotics in the remaining abscess cavity after radical debridement and patch reconstruction of the aortic annulus in patients with aortic valve infective endocarditis seems not to reduce the rate of recurrent endocarditis, reoperation, or mortality.

## Figures and Tables

**Table 1:** Baseline characteristics.

Variable	Local Antibiotic *N* = 20	Control *N* = 21	*p*
**Demographics**			
Age, years	72 (58–76)	68 (62–75)	0.276
Male gender	20 (100)	17 (81)	0.107
Coronary artery disease	12 (60)	7 (33)	0.087
Pulmonary disease	3 (15)	5 (10)	0.697
Dialysis	1 (5)	1 (5)	>0.999
Previous cardiac surgery	11 (55)	14 (67)	0.654
Valve surgery	11 (55)	14 (67)	0.444
Prosthetic valve IE			
Aortic valve prosthesis	10 (50)	14 (67)	0.279
Mitral valve prosthesis	-	1 (5)	0.488
EuroSCORE II	8.4 (2.7–12.2)	9.0 (6.0–20.2)	0.873
**Preoperative clinical status**			
Intubated	-	1 (5)	>0.999
Vasopressor need	-	2 (10)	0.488
Endocarditis-related stroke	7 (35)	7 (33)	0.910
Preserved left ventricular function	16 (80)	16 (76)	0.934
**Inflammatory status**			
C-reactive protein, mg/dL	8.80 (4.55–19.18)	6.50 (1.75–9.50)	0.276
Procalcitonin, ng/mL	0.38 (0.16–1.33)	0.12 (0.04–0.25)	0.128
White blood count, 10^9^/L	7.68 (6.15–14.37)	8.28 (8.28–10.67)	0.873

Data are presented as number (%) or median (IQR); PCI, EuroSCORE, European System for Cardiac Operative Risk Evaluation.

**Table 2:** Microbiological profile.

Variable	Local Antibiotic *N* = 20	Control *N* = 21	*p*
Staphylococcus species	9 (45)	8 (38)	0.654
Staphylococcus aureus	9 (45)	7 (33)	0.444
Streptococcus species	4 (20)	4 (19)	>0.999
Enterococcus species	3 (15)	6 (29)	0.454
Others	1 (5)	1 (5)	>0.999
Negative culture	3 (15)	3 (14)	>0.999

Data are presented as number (%).

**Table 3:** Abscess location.

Variable	Local Antibiotic *N* = 20	Control *N* = 21
NCC	6 (30)	6 (29)
RCC	1 (5)	1 (5)
LCC	1 (5)	2 (10)
NCC-LCC-Commissure	2 (10)	2 (10)
RCC-LCC-Commissure	4 (20)	2 (10)
RCC + LCC	1 (5)	2 (10)
RCC + NCC	1 (5)	1 (5)
Circumferential	3 (15)	2 (10)
AMC	1 (5)	3 (14)

Data are presented as number (%). NCC, non-coronary cusp; RCC, right-coronary cusp; LCC, left-coronary cusp; AMC, aortic-mitral curtain.

**Table 4:** Operative characteristics.

Variable	Local Antibiotic *N* = 20	Control *N* = 21	*p*
Aortic valve replacement	20 (100)	18 (86)	0.261
Biological valve	19 (95)	18 (86)	
Mechanical valve	1 (5)	-	
Aortic valve and root replacement (conduit)	-	3 (14)	0.232
Biological valve	-	2	
Mechanical valve	-	1	
Isolated AV replacement	15 (75)	12 (57)	0.228
Concomitant MV procedure	3 (15)	6 (29)	0.454
Concomitant CABG	4 (20)	2 (10)	0.410
Cardiopulmonary bypass time, minutes	171 (113–219)	130 (120–176)	0.276
Aortic cross-clamp time, minutes	109 (80–144)	101 (88–130)	0.642

Data are presented as number (%) or median (IQR); AV, aortic valve; MV, mitral valve; CABG, coronary artery bypass grafting.

**Table 5:** Endpoints.

Variable	Local Antibiotic *N* = 20	Control *N* = 21	*p*
**Primary**			
Mortality			
30-Day	3 (15)	2 (10)	0.663
2-Year	6 (30)	5 (24)	0.655
Re-endocarditis (2-Year)	1 (5)	1 (5)	>0.999
Reoperation (2-Year)	1 (5)	1 (5)	>0.999
**Secondary**			
Postoperative MCS	3 (15)	4 (19)	>0.999
Dialysis	4 (20)	2 (10)	0.407
Reintubation	3 (15)	6 (29)	0.454
ICU-stay, days	8 (3–12)	8 (5–12)	0.860
Hospital stay, days	15 (8–22)	13 (11–17)	0.399

Data are presented as number (%) or median (IQR); MCS, mechanical circulatory support; ICU, intensive care unit.

## Data Availability

The data underlying this article will be shared on reasonable request to the corresponding author.
